# Cognitive Networks (*Cognits)* Process and Maintain Working Memory

**DOI:** 10.3389/fncir.2021.790691

**Published:** 2022-01-18

**Authors:** Joaquín M. Fuster

**Affiliations:** University of California, Los Angeles, Los Angeles, CA, United States

**Keywords:** cognits, phyletic memory, long-term-memory, perception-action cycle, delay tasks, neuroplasticity

## Abstract

Ever since it was discovered in the monkey’s prefrontal cortex, persistent neuronal activity during the delay period of delay tasks has been considered a phenomenon of working memory. Operationally, this interpretation is correct, because during that delay those tasks require the memorization of a sensory cue, commonly visual. What is incorrect is the assumption that the persistent activity during the delay is caused exclusively by the retention of the sensory cue. In this brief review, the author takes the position that the neural substrate of working memory is an array of long-term memory networks, that is, of cognitive networks (*cognits*), updated and orderly activated for the attainment of a behavioral goal. In the case of a behavioral task, that activated array of *cognits* has been previously formed in long-term memory (throughout this text, the expression “long-term memory” refers to all experiences acquired after birth, including habits and so-called procedural memory, such as the learning of a behavioral task). The learning of a task is the forming of synaptic associations between neural representations of three cognitive components of the task: perceptual, motor, and reward-related. Thereafter, when needed, the composite *cognit* of the task is activated in an orderly fashion to serve working memory in the *perception-action cycle*. To make his points on a complex issue, which has been the focus of his work, and to delineate a frontier for future research, the author refers to several of his own publications and previously published reviews.

## Introduction

Hughlings Jackson (1958) noted that the very same neural elements that *represent* a movement in the motor cortex are in charge of its *execution*. A similar statement can be made on sensation in the sensory cortex with regard to sensory representation and perception. Here I extend that principle to the entirety of the nervous system, from genetic “representations” (*phyletic memory*), like the anatomical structure of primary motor, sensory, and reward systems, to the representation of personal memories in the cortex of association. Memory is recalled or put to work by activation of the neural structure that represents it^1^.

According to this view, a learned delayed-response task, with all its component operations, including working memory, is represented and executed by a vast network of cortical memory that represents sensory stimuli, motor responses, and reward (or approval). Whenever the task requires the mediation of a cross-temporal contingency, as in the delay period, persistent activity links representations of temporally separate task components to bridge time across the contingency between them. If we replace the word *representation* by the word *memory*, in this as in other conditions of the organism, we may reach the conclusion that in the brain there are no systems of memory but there is the memory of systems, and working memory is the temporary activation of perceptual, executive and reward systems’ memory toward a goal.

The interpretation of a cortical cell’s persistent activity as a phenomenon of working memory is entirely in accord with Baddeley’s (1983) basic definition of working memory: the temporary retention of information *for* a behavioral choice or the solution of a problem. Unfortunately, this future aspect of working memory, that is, its “teleonomic” aspect (Monod, 1971), is generally ignored in discussions of persistent activity in the prefrontal cortex.

One clear neural manifestation of the “teleonomic” nature of working memory is the evidence of prefrontal cells whose persistent delay activity is attuned to the animal’s approaching motor response to the cue (Niki, 1974; Quintana and Fuster, 1999). Further, in prefrontal area 8 or its proximity, where a visual directional cue is integrated across a delay with a directional eye movement, persistent delay activity reveals their cross-temporal sensory-motor integration in working memory (Funahashi et al., 1989).

The presence of motion-related neurons in the prefrontal cortex is in harmony with the general notion that this cortex is involved in orderly goal-directed behavioral actions. However, the organization of all such actions requires inputs from sensory areas of the posterior cortex engaged with them in the perception-action cycle. Hence, some prefrontal cells exhibit persistent delay activity that discriminates two stimuli of different modalities—e.g., visual and auditory—if they are associated with each other (behaviorally induced “synesthesia”) across the delay in the performance of a cross-modal delayed matching task (Fuster et al., 2000). Furthermore, in the expectation of a good behavioral outcome or reward, persistent activity can be observed in the orbito-medial areas of the prefrontal cortex (Moorman and Aston-Jones, 2014), which are intimately connected with limbic structures, notably the amygdala and the hypothalamus.

In contrast to the frontal cortex, vigorous sensory-discriminant delay activity can be observed in the temporal (Fuster and Jervey, 1982; Miller et al., 1993) and parietal (Zhou and Fuster, 1996) cortex. Thus we may draw the general conclusion, as others have done (Christophel et al., 2017), that cells in frontal cortices receive multiple sensory and drive-related inputs from posterior sensory cortices and limbic structures for the performance and monitoring of a working-memory task. During the delay, these multiple inputs of diverse origin, which are part of working memory and dispersed in time and cortical space, may average across trials to a semblance of persistent activity. That appearance, however, hides considerable variability from trial to trial (Shafi et al., 2007), as would be expected, though it is not yet proven, from the asynchronous convergence on the prefrontal cortex of task-related inputs from multiple sources, cortical and subcortical. Probably, that prefrontal activity during the delay is driven alternatively by several inputs from the memory of the task, including the cue, the impending motor response, and the expected reward. These inputs from multiple cognitive sub-networks (component *cognits*) upon prefrontal cell populations can be computationally considered and dealt with as *multiple attractors* (Roussy et al., 2021; Wang, 2021).

That cortical inputs are important for the maintenance of working memory and performance of a delay task is evident because the cooling of posterior cortical areas leads to a reversible deficit in the performance of delay tasks and concomitant deficits of activation of frontal cells (Quintana et al., 1989); conversely, the cooling of lateral prefrontal cortex leads to working-memory deficits and disturbance of cell activity in posterior association cortex. Both these phenomena would be manifestations of impaired frontal “cognitive control” (Miller and Cohen, 2001).

The purpose of this review is to emphasize the intimate dependence of working memory from long-term memory and to defend the hypothesis of a common anatomical substrate for both. A related purpose, largely dependent on the validity of that hypothesis, is to defend the corollary that working memory and the persistent neuronal activity that serves it are highly distributed cortical functions of the perception-action cycle.

## Distributed Memory

After the discovery of persistent delay activity as a neuronal manifestation of working memory (Fuster, 1973; Niki, 1974), there was a large number of single-unit studies conducted on primates during the performance of delay tasks. The principal anatomical targets of those studies were the associative areas of the frontal, parietal and temporal cortices. To this reviewer, several general facts became gradually apparent with regard to the task-related cell activity—persistent or not—during those tasks, especially in the light of observations in the human after cortical damage:

(a) In all cortical regions explored with microelectrodes, a large contingent of cells does not alter their discharge in relation to any of the delay-task components. But those cells that do, usually exhibit considerable variability from trial to trial, consistent with temporal variability in the synaptic associative inputs and outputs related to the task. That is also consistent with the expected fluctuations in the perceptual and executive attention (Amengual and Ben Hamed, 2021) devoted by the animal to components of the delay-task habit, which in the trained animal can be safely assumed to be part of long-term memory.
(b) Cue-related activity (sensory) is most prominent in areas of the posterior association cortex, whereas choice-related (motor) activity is most prominent in the prefrontal cortex (especially lateral aspects of it). However, in prefrontal areas heavily involved in sensory-motor integration, such as in haptics (Romo et al., 1999) or in oculomotor behavior (Funahashi et al., 1989), remarkable parametric relationships have been observed between neuronal discharge and stimulus and/or motor response, always within the context of the task previously learned, thus of long-term memory.
(c) The cortical regions from which delay-task activity can be recorded have been implicated by lesion studies in the perceptual or motor memory of the task that the activity is correlated with. For instance, the inferior temporal cortex, from which persistent discriminating cells have been recorded during the delay of visual working memory (Fuster and Jervey, 1982; Miller et al., 1993), has been shown by lesion studies to be a focus of long-term memory of visual discriminations. The same can be said for the posterior parietal cortex with regard to spatial working and long-term memory. Lesions of the prefrontal cortex impair the performance of all delay tasks, as well as of other tasks that, like them, require temporal order of actions and/or the mediation of cross-temporal contingencies (Fuster, 2001). All these tasks are in the long-term memory of the trained animal.
(d) It is in the human brain where, thanks to clinical lesion studies, the most direct relations have been shown between cortical damage and memory deficit (Fuster, 1995, 2009, 2015). Thus, posterior lesions result primarily in deficits of perceptual memory (*e.g.*, agnosias, semantic aphasias, and episodic amnesias), whereas frontal lesions result primarily in deficits of executive memory and functions (*e.g.*, executive neglect, motor aphasia, and problems with executive memory, attention, and planning). In the monkey, lesions of homologs of some of the areas involved in human amnesias and other deficits lead to comparable deficits of perceptual and executive memory, including of course working memory.

The aggregate of these facts provides strong evidence for the following conclusions:

1. All the experimental phenomena of working memory, including persistent delay activity, are phenomena of the processing of the testing task, and therefore of the temporary and orderly activation of the associated components of the long-term memory of the task.
2. Persistent delay activity is an expression of the brain’s necessity to transfer information across time between two or more of those components if they are mutually contingent on one another (perceptual cue, motor choice, and reward).
3. Working memory and long-term memory share the same neural substrate and mnemonic content; working memory is a portion of the long-term memory activated from its resting state and updated in order to mediate cross-temporal contingencies, and thus to conduct the subject to the goal of a task or the approval of the experimenter, or both.

## Cortical Organization of Memory

The facts above support the general principle that working memory consists of an updated cognitive network of long-term memory selectively and orderly activated to attain a goal. Persistent activity is the prime manifestation of it when the attainment of that goal requires the reconciliation of cross-temporal contingencies between associated items of the activated network. It follows that the analysis of the cortical organization of long-term memory should help us understand the neural infrastructure of working memory and its functional dynamics. Here we need a note of caution: the debate about the neural base of memory of any kind or state is often muddled by the assumption of consciousness, ignoring the fact that memory can be unconsciously active and operative.

Because of limits in spatial and temporal resolution, current methods can only provide us with approximate estimates of the cortical regions harboring the highest densities of the most active neural elements—cells and fibers—engaged in the representation of memory, whether this is sensory, motor, emotional, or associative. Those methods, however, are clearly insufficient to define the fine grain of memory and the distribution of specific memories, in other words, what used to be called the “engrams” or “memory traces”. The modern connectome reveals the connective complexity of the cortical substrate of those memories, but cannot tell us about their content any more than a roadmap can tell us about the resources or the economy of a nation. The problem is aggravated by the graded, analog, and probabilistic nature of transactions in the neural cognitive domain. A new paradigm is needed, such as the *cognit* paradigm below, to account for the microstructure and dynamics of cortical memory and cognition.

Nonetheless, as an introduction to the *cognit*, it is useful to consider the general organization of cortical memory at a mesoscopic level, as revealed by the evidence severely summarized above.

Our brain comes to the world with three inherited systems to adapt to it: sensation, motion, and emotion. The *anatomical* *structure* of these three systems, which in life are going to interact intimately with one another, is a form of memory that we all share and that in the course of evolution our species has acquired to deal with the physical and social environments. I call that neural structure of those three systems *phyletic memory* or “memory of the species” because it represents in the form of neural matter, genetically transmitted, the means by which the species in the “night of times” of evolution has acquired (“learned”) to adapt to the environment for subsistence and procreation. Phyletic memory includes the sensory and motor systems and the limbic system, with their peripheral, subcortical, and cortical components. The organism “recalls” and “rehearses” phyletic memory with every sensation, every act, and every emotion.

It is on, and from, the basic grounds of phyletic memory—that is primary sensory and motor cortices, and limbic structures—that all individual memories and knowledge will grow into association cortex to form the long-term memory and habits of the individual organism. Once formed, those memories and habits will be available to be activated *ad hoc* in working memory.

**Figure 1:**
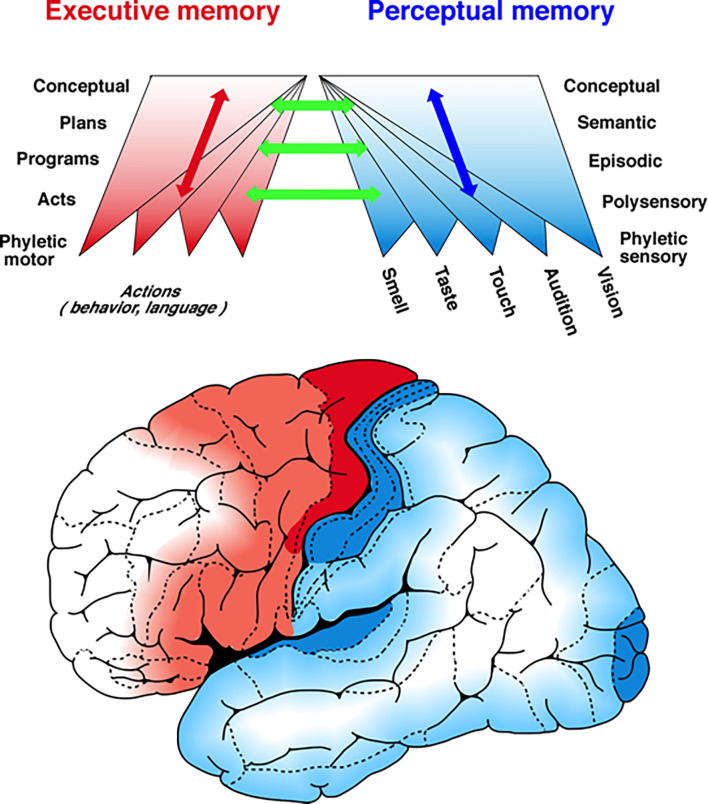
A schema of the hierarchies of cortical memory. The figure portrays in a highly schematic manner a mesoscopic view of the distribution of memory networks. Two hierarchies of memory are shown spread-out tangentially to the cortical surface of the left hemisphere: one in posterior cortex, perceptual, for memories acquired through the senses (*blue to white)* and the other in the frontal cortex, executive, for memories acquired through action (*red to white)*. The two color gradients mark the ascending hierarchies of memory formation and deposition, from the most concrete, sensory- and motor-related, in the lower levels, to the most abstract and complex in the higher levels of both hierarchies. Up each hierarchy, as memory networks accrue and find their hierarchical niche, they connect with preexisting ones at every level by reasons of similarity and common features. As the upper figure implies, the networks of memory and knowledge (semantic memory) are formed by convergence as well as divergence of associative connections. The two bi-directional arrows, one blue and the other red indicate not only the ascending and descending connections within each hierarchy but also the heterarchical connectivity in some memories and their networks. The green arrows symbolize the connections between hierarchies that play such a critical role in the perception-action cycle. From Fuster (2015).

In a comprehensive review of primate single-unit studies of working memory in the visual system of primates, Roussy et al. (2021) arrive at conclusions that remarkably support the ideas expressed above about the hierarchical organization of areal memory networks. The authors conclude as I predict from my studies, that beginning with the striate cortex (“phyletic memory”), successively higher areas in a hierarchy that reaches the prefrontal cortex engage progressively less in “perception” and more in executive memory. I would object to the use of the word “perception” instead of *sensation* (perception is already individual memory), but the principle is valid: by ascending the visual hierarchy, vision becomes more memory, perceptual memory that is, and therefore more specific to the individual. This is an argument for the increase and expansion of idiosyncratic connectivity up the hierarchy (symbolized by the upward diverging cones in Figure 1). It is also an argument for a common substrate for working and long-term memory.

## The Cognit

The idiosyncrasy of personal memory can only be understood by the combinatorial power of some 20 billion cortical neurons and their connections. To understand the microstructure of memory, its widespread roots and branches throughout the cerebral cortex, as well as its dynamics in retrieval and working memory, we must construct a new paradigm based on two fundamental principles of neurobiology that apply to all levels of neural cognition, from phyletic to semantic memory:

(a) Every sensation and every movement defines itself, and acquires neural function and meaning, in relation to other stimuli or movements that have been apprehended or learned together with it, whether in evolution or in the life of the individual organism. At the evolutionary level, the “elementary sensation” (Mach, 1885) does not exist (Hayek, 1952), because every sensory feature, however simple, has some spatial or temporal dimension and continuity into itself. The neurocognitive code is basically a *relational code* and all memory is associative, even at the level of the neuronal columns or groups of neurons that represent minimal sensory or motor features. Excitation and inhibition—e.g., in the retina or in antagonistic muscles—provide strength and contrast to each other: this is true, for example, in the flexors and extensors of the leg, whether in its innate defensive leg withdrawal or in normal walking. Context and background provide essential associations to define the memory of a stimulus or a movement.
(b) Even at rest, the connectivity of a cortical memory network, which links neuronal columns or groups together, is never static. Synaptic weights change with general metabolism, circadian rhythm, developmental stage, and age. The synaptic connectivity changes increase markedly with reactivation of the network in retrieval, new learning, or new experience. More generally, abrupt synaptic changes occur by engagement of the network in any kind of sustained cognitive operation, such as working memory or consolidation. All these changes take place in large and specific memory networks that join widely separated cell groups in the cerebral cortex.

Most empirical or computational models of cortical memory ignore those two principles and, in addition, the growing evidence that memory networks serve not only memory operations but also the other cognitive functions: attention, perception, language, and intelligence (Fuster, 2003). Memory, of any kind, is the essential neural substrate those functions operate on and with. Our attention, both serial and parallel, is guided by memory. In perception, we project memory on the environment, “we not only remember what we see but see what we remember” (Helmholtz, 1860). Language is essentially based on semantic memory. Intelligence makes use of all of the above plus executive memory.

It is the evidence that memory networks are the basic neural units *of all* cognitive functions that led me to the *cognit paradigm* and to rename those networks *cognits*. The new paradigm is founded on a new conceptual methodology to approach the cognitive brain, the knowing and remembering brain. Its principal new feature is a Copernican shift of the basic cognitive unit from the neuron or cortical area to the widely distributed network of cortical neurons, where association, connection, and relationship define structure and mechanisms at the microscopic level within widely distributed networks.

The cortical cognitive networks that I propose are considerably different from those in the available literature. Regardless of their empirical or theoretical base, those published networks ordinarily link together anatomically or physiologically defined areas of the cerebral cortex. Instead, my postulated networks link neuronal groups within and between multiple cortical areas, some of those groups widely separated. Here are some of its distinguishing features:

1. A *cognit* consists, in and of, a net of cortical nerve cells and the fibers and synapses that unite them. That structure contains in itself an item of memory or knowledge acquired by life experience. Cognits are exquisitely idiosyncratic, specific for each individual, differing in location, extension, and synaptic strength, depending on such factors as age, experience and training, or education.
2. The anatomical outlines *of a cognit* are diffuse and highly irregular, as it blends at its margins onto other associated cognits with weak or unstable connections. Depending on their synapse and fiber complexity, *cognits* vary considerably in size and cortical coverage. Because they share cell groups and connections representing common associated features, cognits interconnect and overlap profusely with one another.
3. A *cognit* develops out of phyletic memory—primary sensory or motor cortex—and into the associative cortex in accord with Hebbian principles (Hebb, 1949), by associations of spatial and temporal coincidence between new sensory and/or motor—proprioceptive—stimuli. In addition, those stimuli can activate, and establish connections with, pre-existing and related *cognits*, to form with them more complex cognits, thus expanding prior memory and knowledge. This will occur under inputs and influences from the hippocampus (archicortex)—by still unclear mechanisms—and the amygdala, the latter contributing emotional connotations to the new or updated cognits.

As they develop in the course of life, the new and expanded *cognits* will occupy progressively larger and hierarchically higher areas of the associative cortex, while retaining connections with lower, nested *cognits*. Because of the unlimited possibilities of connection (combinatorial power) between cortical neurons, and because of the graded (not “all-or-none”) strength of their synaptic interconnections, the higher generated *cognits* are profusely distributed over cortex, overlapping and interconnecting with one another and with the lower ones nested within them.

By virtue of the practically infinite possibilities of interconnection between cortical cell groups or modules to form a *cognit*, and the interactions between cognits, the size, location, and synaptic stability of a given cognit varies greatly over time. Plasticity under personal experience, attrition with time and age, and anatomical overlap is the norm for all *cognits* and what gives them individuality. Furthermore, because of interactions and overlaps, any cell or group of cells practically anywhere in the association cortex can be part of *many cognits*, thus many memories or items of knowledge.

The new paradigm does not supplant more conventional memory networks linking cortical areas, but it complements them with greatly magnifying “optics”. Under their view, the *cognit* is individualized, much more extensive and intricate than areal networks, and it serves not only memory but also the other cognitive functions as well; hence the word *cognit*.

Recently, Fulvi Mari (2021) has published a computational model of memory retrieval in a modular associative network with an architecture extraordinarily similar to that here postulated for the *cognit*. The model suggests storage and retrieval mechanisms across different levels of a memory hierarchy of networks.

Our views of memory leave little room for the traditional classes of memory (episodic, declarative, implicit, etc.) and even less room for their anatomical location. Nonetheless, as indicated in previous sections, there is now sufficient evidence from humans and monkeys to roughly trace the cortical paths of formation of the various *cognits* and the approximate anatomical location of their foci (nodes) of heaviest associations.

In recent years, physiological animal studies have confirmed the upward trend toward higher categories of *cognits* in the perceptual hierarchy that has long been recognized by clinical studies in the human brain. That trend culminates in the prefrontal cortex, where the highest-order sensorimotor *cognits* and integrations take place (Brincat et al., 2018; Reinert et al., 2021).

Connection fibers ascending the two hierarchies, perceptual and executive, from area to area, are reciprocated every step of the way by fibers running in the opposite direction (Figure 1). Some fibers descend directly (through the lateral longitudinal fasciculus) from the prefrontal cortex to the cortex of association beyond sensory cortices (*e.g.*, the inferior temporal cortex). These fibers evidently engage in what has been called “cognitive control” (Miller and Cohen, 2001; Goodwin et al., 2012). Cognitive control is exerted over working memory networks and generates in them persistent activity when there are discontinuities in the perception-action cycle.

## Working Memory in The Perception-Action Cycle

**Figure 2:**
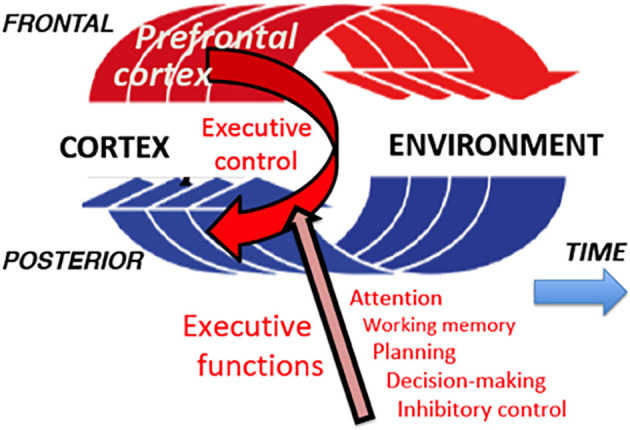
Perception-action cycle. In a sequence of goal-directed actions, each action causes a change in the environment, which generates sensory impulses; these impulses are analyzed in the posterior association cortex (in perceptual long-term memory), and the result of this analysis informs the frontal cortex (executive long-term memory) for the next action. And so on and so forth, cycle after cycle, until the behavioral goal is reached. At every turn of a cycle, the prefrontal cortex matches percept and action to the long-term memory of both in the present context, and exerts updated executive control of them through its executive functions.

The perception-action cycle is the ultimate evolutionary development into the cerebral cortex of the innate systems and mechanisms of the organism to adapt to changes in the internal and external milieus (Uexküll, 1926). The internal milieu is stabilized by the autonomic nervous system and neuroendocrine systems (homeostasis). For adaptation to the external environment, the organism is born with an array of reflex arcs in the spinal cord and mesencephalon that serve it to satisfy immediately vital needs and may be considered part of phyletic memory. At the level of the cerebral cortex, the cortical regions for adapting cognitive behavior to the physical and social environments constitute the highest substrate of the perception-action cycle. In the aggregate, this substrate forms a highly plastic and versatile system of adaptation. It is a biocybernetic system with feed-forward and feedback that governs cognitive interactions of the organism with the exterior, including such high cognitive functions as is conversational language (Figure 2).

In order to understand the physiological functions of the cycle, especially the role of the prefrontal cortex in it, and persistent activity in its neural circuitry, it is useful to consider certain general assumptions that derive from the human brain (Fuster, 2015):

1. The cortex of the frontal lobe is essential for the temporal organization of orderly behavior toward a goal, especially if the behavior and the goal are novel (Luria, 1966).
2. To that effect, the cortex of the frontal lobe necessitates subcortical inputs of drive and motivation, as well as the basal ganglia as outputs to accion, together with the pyramidal system.
3. The cortex of the frontal lobe, especially the lateral and medial prefrontal cortex, has a predictive, anticipatory property that allows the organism to become future-oriented, error-predictive, and “pre-adaptive.”
4. The prefrontal cortex, especially its orbital region, is important for inhibitory control of distractions by interfering stimuli, impulses, and memories.

None of these global functions of the human frontal cortex is strictly specific for any frontal region in particular, and there is considerable individual variability in the dominance of any of them in any particular region. There is, however, a group of functions best identified in the prefrontal cortex of the nonhuman primate, which is somewhat topologically related to those of the human and that serves the perception-action cycle in the temporal organization of behavior. These functions (listed on the lower right of Figure 2) are the so-called executive functions of the prefrontal cortex, grouped under the heading of *executive control*.

Note that the first in the list is attention, a cognitive function—*not necessarily conscious*—which supports all other executive functions and consists in selectively allocating to them the limited neural resources available. The second executive function is working memory, so dependent on attention that Baddeley (1993) was inclined to consider it attention to an internal representation. The third function, planning, is attention directed to future actions, including attention to the preparation of actions in the short term. Decision-making is selective executive attention by definition. Finally, inhibitory control is also an aspect of attention, by definition, that is, the exclusionary form of attention: it is the inhibition of any source of interference, internal or external that might impede the perception-action cycle to attain its goal.

In the temporal course of the perception-action cycle toward that goal, the focus and content of attention and the role of the prefrontal executive functions over posterior—perceptual—cortical regions shifts, within the present context, from one item in long-term memory to another—updated to the present. That long-term memory can be, for example, the performance of a delay task. Naturally, in the case of a trial of such a task, the items that will attract the most attention in a given trial will be the sensory cue and the motor response, both of which will be novel *for that trial*.

The perception-action cycle can be set into motion in any of its compartments, internal or external. Examples of cycle starters would be an internal plan with a long-term objective, an emotional encounter, a biological urge, a sensory experience, or a combination of any of them. The cycle circulates through cortical memory, perceptual and executive, and through the environment. Cycle after cycle, with changing input and output, though with a consistent goal, the perception-action cycle epitomizes what could be characterized as the adaptive dynamic infrastructure of the cortex.

Some parts of the cycle that are constant and repetitive, such as the habitual actions in every trial of a delay task, circulate through the cortex and, in addition, through subcortical reflex arcs, including the basal ganglia (Daw et al., 2005). The task itself is represented by a high cortical *cognit* and its parts by nested subordinate *cognits* at lower hierarchical levels. All are sequentially recruited and activated under the cognitive control of the prefrontal cortex, which ensures order and guidance to the sequence. But the sequence of active *cognits* is essentially self-generated and self-organized by association. Thus the cognits were initially formed by association, and now by association are sequentially activated in the perception-action cycle. Accordingly, the perception of the cue at the beginning of a delay trial is an act of *recall*, which by association will evoke successive cycles in every trial to attain its reward.

In the enforced delay of any delay task, the short-term memory of the cue and the prospective memory of the response will dominate, the first in sensory association cortex and the second in the prefrontal cortex, both probably maintained and mutually reinforced by reverberating activity between the active cognits of the two cortical regions. Those activated *cognits* will be the two main sources of persistent activity in those cortical regions.

**Figure 3:**
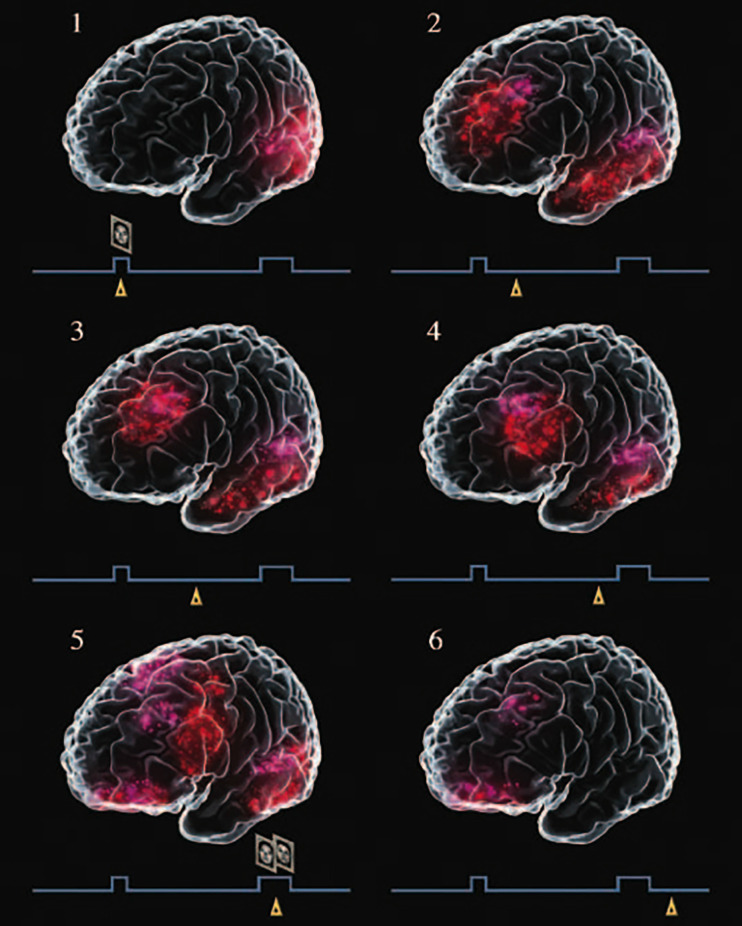
Graphic meta-analysis of cortical activations in the course of a delayed matching-to-sample task, paradigmatic of visual delay tasks in a large number of functional neuroimaging studies. A trial begins with the presentation of a face (sample), which the subject must remember for a delay of 10–15 s, at the end of which the subject is presented with two faces and must choose the sample. Little triangles mark the approximate relative timing of the records in the course of a trial. Note the activation of the visual cortex at the sample (excerpt 1), and of the inferior temporal and prefrontal cortices during the delay (excerpts 2–6). From Fuster (2015).

Figure 3 is the result of a *graphic meta-analysis* of a large number of functional neuroimaging studies of human subjects performing visual delay tasks^2^, thus in the perception-action cycle. During the delay, when persistent activity—in averages or single trials—is most likely to occur, activation is seen simultaneously in visual association cortex and lateral prefrontal cortex. As the delay progresses, the prefrontal activation grows and advances toward the motor cortex, anticipating the choice-response. The joint activation of the prefrontal and infero-temporal components of the network representing the task, with their loop of persistent activity, serves as a bridge of working memory at the top of the perception-action cycle.

Ascribing to the prefrontal cortex the “seat” of executive control with its five executive functions (Figure 2) is supported by a massive amount of data. However, this fact may lead to the mischaracterization of the prefrontal cortex as the “central executive” or the “center of will”. It is neither, even though it mediates executive functions, free choice, and creativity. Indeed, to give to our prefrontal cortex the role of the autonomous origin of all our decisions and actions leads inevitably to an infinite regress that should be avoided (“What agency controls the prefrontal cortex? What other agency controls that one?...and so on *ad infinitum*). The only reasonable solution to the quandary is to place the prefrontal cortex in the perception-action cycle, where the action can originate anywhere, including the cerebral cortex, prefrontal or other.

Thus the prefrontal cortex does not escape Jackson’s principle: the same neural structure harboring the memory of an action is in charge of its execution. So, we have to pose ourselves two questions: what kind of memories does the prefrontal cortex hold for the long-term? What kinds of cognitive networks does it hold that, under certain circumstances, can become executive networks by entering the perception-action cycle? The answer to both questions seems to be that the prefrontal cortex contains memory networks (*cognits)* of plans of action or a series of goal-directed actions, ready to be activated in working memory. Here we are adding an essential parameter of prefrontal memories and *cognits*: time (Fuster, 2001). Associations of timing and order encoded in those networks, pace and time the executive functions that lead those activated and operational networks to their objective. What’s more, the prefrontal cortex can create within itself new *cognits*, new memories out of old ones, thus predicting, imagining and creating future actions (Ingvar, 1985; Addis et al., 2007; Fuster and Bressler, 2015), in addition to bridging cross-temporal contingencies with working memory and with persistent activity of its cognitive neuronal networks or *cognits* of the cerebral cortex at large.

Although it can evoke and create a new action, the prefrontal cortex cannot execute it without the intimate cooperation of the other cortical and subcortical participants in the perception-action cycle. A suitable analogy for that cortex would be that of both composer of the music and director of the orchestra.

## Discussion

The *cognit* paradigm would have to be rejected if it were shown by reliable methods that a personal memory, a percept, or a sequence of organized action were localized in its entirety in a discrete portion of the cortex. Such evidence would negate the essentially distributed character of a *cognit* and working memory, in addition to the critical phenomenon of perceptual constancy (“a rose is a rose, is a rose,” regardless of size, color, aroma, or position in my visual field).

The new paradigm stems in part from the failure of all forms of cerebral *localizationism* of memory. Also, it is an attempt to substantiate by neuroscientific methodology four classic theories of cognition: associationism, Gestalt psychology, connectionism, and the Cajal-Hebb synaptic theory. Despite their shortcomings, all four theories share important properties with the *cognit*, and therefore, of the substrate of working memory I postulate.

Associationism, the psychological doctrine introduced in ancient times by Aristotle and widely advocated by British empiricists (17th–18th centuries), reduces all mental life to associations between mental states, ideas, sensations, reflexes, etc.., dividing the mind into components but ignoring its unity and the functional relations between those components. One exceptionally useful concept of associationism is that of the association between sensation and memory in perception (“we remember what we see, and see what we remember”) With regard to memory, associationism fails to recognize its hierarchical organization, and of course, there is no place in it for heterarchical associations.

Associationism and connectionism clearly accommodate the concepts of synapsis and neural network that serve cognition in the brain. Any neurophysiological analysis of cognition based on them, however, must deal with neural signals that are for the most part analog and probabilistic, like firing frequency and field potentials. These are not the most convenient signals for models and machines in the field of artificial intelligence. Nonetheless, connectionist neural-network algorithms applied to language can discover certain categories of grammatical rules based on similarities, much as the *cognit* paradigm can uncover hierarchical categories of language (McClelland and Rumelhart, 1986).

Gestalt psychology (Koffka, 1935) provides the most proximate property to semantic memory, and with it to high-category *cognits*: an object is defined by the relations between its parts, not by the parts themselves, and certainly not by the sum of those parts. However, as in the case of connectionism, the meaning of a Gestalt (“structure”) lies in both the relation and the related elements. Given the practically infinite combinatorial power of over 20 billion neurons, the potential variability of human memories is immense, like that of human experience. There are, however, constraints to that variability dictated by anatomy and physiology. One is the innate connectivity of the individual brain. Another is the strength of synaptic connections. These constraints exist at all levels of the cognitive hierarchy, but are presumably more stringent at their higher levels, where semantic knowledge and global action are constituted by convergent affluences from lower, nested, and more concrete *cognits*. Hence, by assumed foci of synaptic strength and fiber convergence, it seems legitimate to grossly delineate the relative position of the various categories of knowledge and memory on the cortical surface (Figure 1).

All three psychological theories mentioned in support of the *cognit* paradigm have the most reasonable neurobiological foundation in the synaptic principles of memory formation first proposed by (Cajal, 1894) and (Hebb, 1949). These principles culminate with the idea of the “neuronal assembly,” which is the theoretical precursor of the *“cognit”*, though the latter applies to all cognitive functions, not just memory. Further, with regard to memory, Hebb’s concepts are based on circuitry mostly circumscribed to the visual and the parastriate cortex, whereas the *cognit* extends to association cortex of all sensory and motor systems. Both conceptions, Cajal’s and Hebb’s fail to explain the role of the hippocampus, decisive but still poorly understood till now, on the formation of new neocortical memory.

In recent years, the *cognit* paradigm has found some support in the latest investigations of cortical connectionism with the most advanced techniques available. Among the latest initiatives based on those techniques is the *connectome*, the international research program to expose the entire connectivity of the human brain. This effort has led to exquisite maps of cortical connectivity, spectacular for its richness, but so far it has not helped us much to reveal cortical neuroplasticity, one of the objectives of the program. Functional resting-state magnetic resonance imaging (fMRI) is another promising method (Taren et al., 2011).

Future research should be devoted to obtaining better spatial and temporal resolution than we now have of cerebral processes in active memory. The dependency of working memory from long-term memory could be supported by utilizing—in working memory tasks—stimuli (cues) that activated different levels of the memory hierarchy. The critical question would be if the same cortical hierarchy of memory would be evinced by simple recall as by working memory of sensory stimuli of differing levels. This would further confirm my parsimonious proposal of an identical neural substrate for both conditions of activated memory.

Another issue for future research is the predictive and prospective executive functions of the prefrontal cortex, such as planning, executive attention, and working memory, in the acquisition of memory and knowledge. Training children in those functions is the key to the success of *active learning*, the educational method that capitalizes on the initiative, creativity, and cooperativeness of the child. This method is at the foundation of the most successful modern systems of elementary education, such as the Finnish system.

## Conclusion

The presence in the primate brain of a system for long-term memory and another for working memory is at odds with all the pertinent empirical evidence. Instead, a massive body of experimental and clinical evidence indicates that working memory consists of the temporary activation of an updated cortical network of long-term memory for the attainment of an objective. That accords with the general principle of this review: under appropriate circumstances, any memory of the organism, from the biological to the most abstract, can become operational in behavior, reasoning, and in the spoken or written language. Working memory is operational memory by definition and the epitome of that principle. Its most elementary substrate is a cortical network of long-term memory, here called *cognit*, formed between neurons by associations according to Hebbian principles. A *cognit* is specific for a given individual; in working memory, it is updated for present context The dynamics of working memory can best be examined and understood in the perception-action cycle, the biocybernetic loop that engages the organism with its environment in goal-directed behavior. Working memory bridges with persistent activity in widely distributed cortical networks any temporal break or discontinuity that may occur in the cycle before reaching its goal.

## Author Contributions

The author confirms being the sole contributor of this work and has approved it for publication.

## Conflict of Interest

The author declares that the research was conducted in the absence of any commercial or financial relationships that could be construed as a potential conflict of interest.

## Publisher’s Note

All claims expressed in this article are solely those of the authors and do not necessarily represent those of their affiliated organizations, or those of the publisher, the editors and the reviewers. Any product that may be evaluated in this article, or claim that may be made by its manufacturer, is not guaranteed or endorsed by the publisher.

## Footnotes

^1^The analogy with immune systems is remarkable. “Memory T-cells” are characterized by their long immunological memory.

^2^Because of the limitations of neuroimaging, especially in temporal resolution, as well as differences in the time scales of the studies analyzed (some of them meta-analyses of others), the time course of activations has been estimated from unit studies in the monkey. Because of the large number of studies analyzed, the problem of reverse inference (Poldrack, 2006) is presumably avoided.
